# White light emitting diode induces autophagy in hippocampal neuron cells through GSK-3-mediated GR and RORα pathways

**DOI:** 10.18632/aging.101878

**Published:** 2019-03-28

**Authors:** Yang Yang, Yimin Jia, Qinwei Sun, Haibo Dong, Ruqian Zhao

**Affiliations:** 1 MOE Joint International Research Laboratory of Animal Health and Food Safety, College of Veterinary Medicine, Nanjing Agricultural University, Nanjing 210095, P. R. China; 2 Key Laboratory of Animal Physiology and Biochemistry, Nanjing Agricultural University, Nanjing 210095, P. R. China

**Keywords:** White LED light, autophagy, GSK-3, Glucocorticoid receptor, RORα

## Abstract

Autophagy plays an important role in cell survival under diverse stress conditions. Here, we show that white LED light exposure for 24 h significantly activated autophagy-related genes and increased autophagosome formation in hippocampal neural cells (HT-22). Concurrently, the rhythmic pattern of clock-related gene expression was disrupted, which was associated with augmented expression of SIRT1, AMPK and retinoid-related orphan receptor alpha (RORα). SR1001, a specific inhibitor of RORα, protected the cells from light-induced activation of autophagy. Moreover, light exposure increased glucocorticoid receptor (GR) phosphorylation and nuclear translocation. GR inhibitor RU486 prevented light-induced up-regulation of RORα and the activation of autophagy. These changes were associated with enhanced glycogen synthase kinase-3 (GSK-3) activity and its specific inhibitor CHIR-99021 significantly rescued light-induced autophagy and augmented GR, RORα and autophagy-related proteins. Furthermore, GSK-3 was identified as an upstream regulator of GR/RORα signaling as it was not affected by GR or RORα inhibitors. Taken together, our data demonstrate that GSK-3-mediated GR/RORα signaling pathway is involved in white LED light-induced autophagy in hippocampal neuron cells.

## INTRODUCTION

Autophagy is a process that disassembles unnecessary or dysfunctional cellular components for degradation and recycling [1]. The formation of autophagosome, a double-membraned vesicle that eventually fuses with lysosomes, is a critical step in macroautophagy. Autophagy-related genes (ATGs) family and microtubule-associated protein 1A/1B-light chain 3 (LC3) are crucial regulators in autophagosome formation [2]. The cytosolic form of LC3 (LC3-I) is conjugated to phosphatidylethanolamine to form LC3-II, which is recruited to autophagosomal membranes. After fusion of autophagosome with lysosomes, LC3-II on the cytosolic face of autophagosomes is delipidated by Atg4B to form LC3-I for recycling, and LC3-II on the lumenal face of autophagosomes is degraded by lysosomal hydrolases [3]. Therefore, LC3-II is used as a promising autophagosomal marker to reflect autophagic activity. Autophagy is a double-eged sword in cell function [4]. It may promote cell survival through inhibition of apoptosis [5] or induce cell death through caspase and apoptosis-independent mechanisms [6]. Oxidative stress has been shown to cause the accumulation of autophagosomes in different types of somatic cells [7]. Various conditions that induce oxidative stress, such as starvation [8], infection [9], and environmental stress [10], can activate autophagy. Excessive light exposure has been shown to induce autophagy in different types of cells. Constant UV light exposure for 24 h activates autophagy in epidermal cells [11]. Near-infrared photothermal therapy increases autophagic cell death in breast cancer cells [12]. Light emitting diodes (LEDs), with superior efficiency, lower cost and high variability of wavelengths ranging from the ultraviolet to the near-infrared region of the spectrum, have been developed to replace traditional light bulbs. LED light exposure has been reported to cause cell damage through activation of autophagy in colon cancer cells [13], retinal pigment epithelium cells [14], retinal photoreceptor cells [15] and lymphoid cells [16]. We reported previously that white LED light exposure induces apoptosis and cell cycle arrest in hippocampal neuron cells [17]. However, it remains unknown whether LED light may activate autophagy in hippocampal neuron cells.

The activation of autophagy is regulated by complex intracellular signaling networks. Glucocorticoid receptor (GR) is highly expressed in hippocampus to mediate the central response to peripheral glucocorticoids under basal and stressful situations [18]. Glucocorticoids are reported to induce the autophagic processes in N1511 chondrocyte cells [19] and rat bone marrow mesenchymal stem cells [20]. Also, a ligand-independent activation of GR is involved in the transcriptional regulation of autophagy-related genes in HT-22 hippocampal neuron cells [21]. Autophagy flux is known to have circadian rhythms [22], while circadian clock genes, which are regulated by transcription factors such as retinoid-related orphan receptor alpha (RORα) [23, 24], are reported to regulate autophagy [25]. Moreover, the serine/threonine kinase glycogen synthase kinase-3 (GSK-3) plays a paradoxical role in autophagy. Inhibition of GSK-3β suppresses autophagic cell death in adult hippocampal neural stem cells [26], whereas inhibition of GSK-3 activates autophagy in human pancreatic cancer cells [27], breast tumor cell line MCF7 [28], and prostate cancer cells [29]. Although the role of GSK-3, GR and RORα in the regulation of autophagy has been reported respectively, it remains unknown whether and how they are involved in mediating the effects of white LED light on hippocampal neural cell autophagy.

In this study, we first show that LED light exposure significantly activated autophagy and disrupted the circadian rhythms of clock related genes in hippocampal neuron cells, which was associated with significantly increased GSK-3, phospho-GR and RORα protein expression. To further delineate the signaling pathway of LED light-induced autophagy in hippocampal neuron cells, we used inhibitors or shRNAs to specifically knockdown RORα, GR and GSK-3, respectively. The results indicate that GSK-3-mediated GR and RORα pathways participate in the regulation of the autophagy activation in white LED light-exposed hippocampal neuron cells.

## RESULTS

### White LED light exposure induces autophagy in hippocampal neuron cells

**Figure 1:**
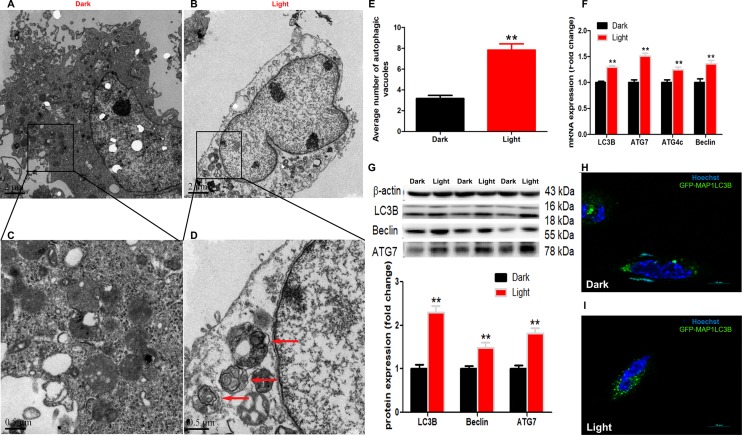
**White LED light induces activation of autophagy process in HT-22 cells.** (**A**–**B**) TEM images of autophagic vacuoles in Dark and Light groups, respectively. Scale bars, 2 μm, n = 3; (**C**–**D**) Higher magnification of indicated regions in A and B (squares), showing autophagosomes with double membrane (red arrows). Scale bars, 0.5 μm; (**E**) The number of autophagic vacuoles. Values are means ± SEM, ** *p* < 0.01 compared with Dark group, n = 3. (**F**) Quantitative Real-time PCR analysis of autophagy-related gene *lc3b*, *atg7*, *atg4c* and *beclin.* Values are means ± SEM, ** *p* < 0.01 compared with Dark group, n = 6; (**G**) Western blot analysis of autophagy-related protein LC3B, Beclin and ATG7. Values are means ± SEM, ** *p* < 0.01 compared with Dark group, n = 6; (**H**–**I**) Representative fluorescence images of HT-22 cells transfected with GFP-LC3 plasmid. Cells were counterstained with Hochest (nuclei in blue) and more autophagosomes (green) were seen in Light group. Scale bar, 10 μm.

White LED light-induced autophagy in hippocampal neuron cells was demonstrated by using different methods. Firstly, transmission electron microscopy (Figure 1A–1D) demonstrated that the number of autophagic vacuoles was significantly increased (*p* < 0.01) in Light group (Figure 1E). Secondly, autophagy-related genes, such as LC3B, Beclin and ATG7, were significantly (*p* < 0.01) up-regulated in Light group at both mRNA (Figure 1F) and protein (Figure 1G) levels. Thirdly, the GFP-LC3B immuofluorescence was significantly increased (Figure 1H–1I) in light-exposed cells transfected with the GFP-tagged MAP1LC3B expression plasmid (GFP-MAP1LC3B). Furthermore, blocking the autophagy with 3-MA, an early stage autophagy inhibitor, rescued light-induced decrease of cell viability, in a dose-dependent manner (Supplementary Figure 2).

### White LED light exposure enhance autophagy flux in hippocampal neuron cells

**Figure 2:**
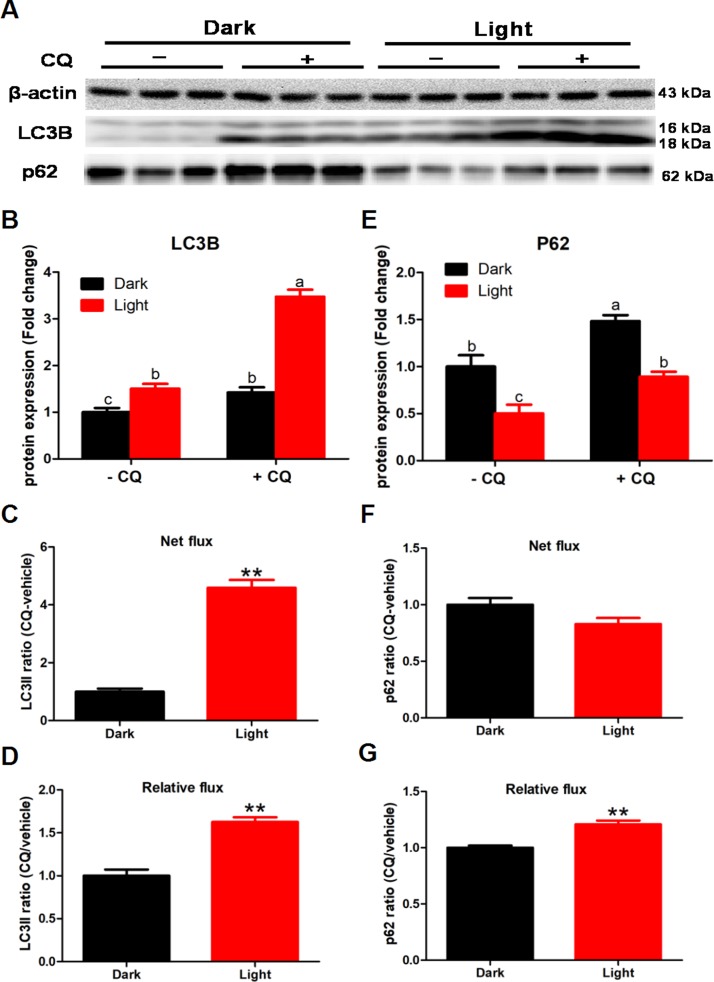
**White LED light exposure enhance autophagy flux in hippocampal neuron cells.** To detect the effect of white LED light on autophagy flux, we added 50 μM chloroquine (CQ) in the last 2 h of white LED light exposure. (**A**) Images of bands detected in Western blot analyses; (**B**) LC3B protein levels in Dark and Light group with or without CQ. Values are means ± SEM. Bars with different superscripts are significantly different from each other (*p* < 0.05, n = 3); (**C**) Net flux of LC3II protein. Values are means ± SEM, ** *p* < 0.01 compared with Dark group, n = 3; (**D**) Relative flux of LC3II protein. Values are means ± SEM, ** *p* < 0.01 compared with Dark group, n = 3; (**E**) p62 protein levels in Dark and Light group with or without CQ. Values are means ± SEM. Bars with different superscripts are significantly different from each other (*p* < 0.05, n = 3); (**F**) Net flux of p62 protein. Values are means ± SEM, n = 3; (**G**) Relative flux of p62 protein. Values are means ± SEM, ** *p* < 0.01 compared with Dark group, n = 3.

To detected the effect of white LED light on autophagy flux, we added 50 μM chloroquine (CQ) in the last 2 h of white LED light exposure. CQ significantly increased LC3II and p62 protein levels (*p* < 0.01) both in Dark and Light group (Figure 2B and 2E). Meanwhile, white LED light exposure significantly increased (*p* < 0.01) LC3II flux both in net flux and relative flux (Figure 2C and 2D). Concurrently, white LED light exposure significantly increased (*p* < 0.01) the relative flux of p62 (Figure 2G), yet not influence net flux of p62 (Figure 2F).

Surprisingly, we found that different CQ added mode lead to different autophagy flux results. LC3II and p62 protein levels were significantly increased (*p* < 0.01) both in Dark and Light group with added 50 μM CQ in 2 h after white LED light exposure (Supplementary Figure 3B and 3E). However, white LED light exposure significantly decreased LC3II flux both in the net autophagy flux (*p* < 0.01) and the relative autophagy flux (*p* < 0.05) (Supplementary Figure 3C and 3D). Concurrently, white LED light exposure significantly increased (*p* < 0.05) the relative flux of p62 (Supplementary Figure 3G), yet not influence the net flux of p62 (Supplementary Figure 3F).

### White LED light activates GSK-3 and nuclear receptors GR and RORα in HT-22 cells

**Figure 3:**
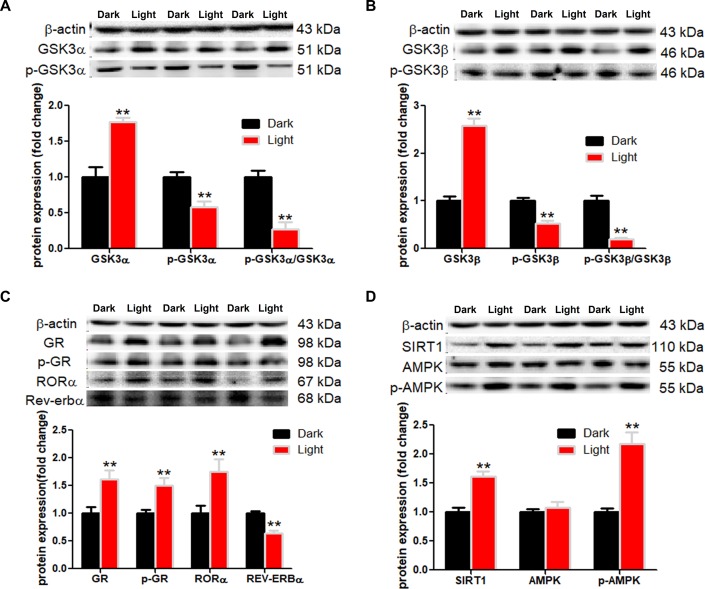
**White LED light activates GSK3 and nuclear receptor GR, RORα in HT-22 cells.** (**A**–**B**) Protein content of total and phospho-GSK-3α/β (Ser21/9). Significant decrease of p-GSK3/GSK3 ratio in Light group indicates activation of both GSK3α and GSK3β; (**C**) Protein content of GR, phospho-GR, RORα and REV-ERBα, showing up-regulation of both GR and RORα in Light group; (**D**) Protein content of SIRT1, AMPK and phospho-AMPK. Values are means ± SEM. **p < 0.01, compared with Dark group (n = 6).

White LED light exposure significantly increased protein levels of GSK-3α and GSK-3β (*p* < 0.01), yet the serine-21 phosphorylated GSK-3α (Figure 3A) and serine-9 phosphorylated GSK-3β (Figure 3B) were significantly decreased (*p* < 0.01), indicating enhanced GSK-3 activity as phosphorylation at these particular serine residues inhibits the activity of GSK-3α and GSK-3β, respectively [30]. Concurrently, protein contents of GR and phospho-GR were also significant increased (*p* < 0.01). Moreover, RORα was significantly increased (*p* < 0.01), while REV-ERBα significantly decreased (*p* < 0.01) in light-exposed cells (Figure 3C), implicating possible disruption of clock genes and their circadian rhythms. Furthermore, SIRT1/AMPK pathway, which is known to regulate autophagy, is activated in response to white LED light exposure. The protein levels of SIRT1 and phospho-AMPK were significantly increased (*p* < 0.01) after LED light exposure (Figure 3D).

### White LED light disrupts the circadian rhythm of circadian clock genes in HT-22 cells

**Figure 4:**
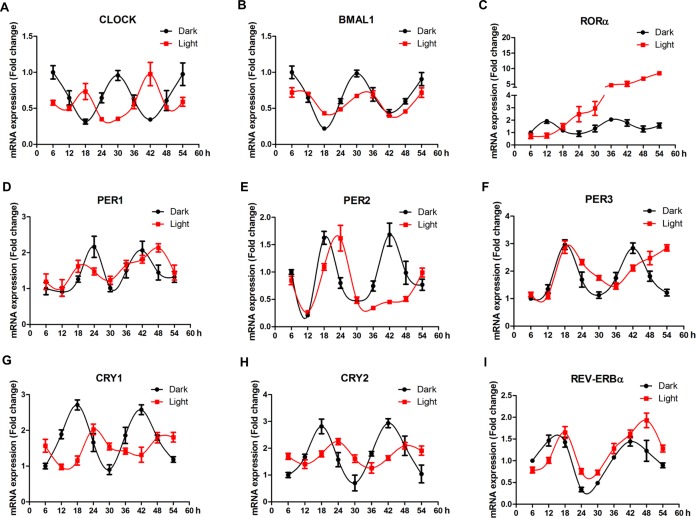
**White LED light influences the circadian rhythm of clock-related genes in HT-22 cells.** (**A**–**B**) *Clock* and *bmal1* mRNA, showing time-phase shift and suppressed oscillation amplitudes in Light group; (**C**) *Rorα* mRNA, showing diminished circadian rhythm and a continous increase in mRNA abundance over the period of examination in Light group; (**D**–**E**) *Per1*, *per2* and *per3* mRNA expression, showing reduced oscillation frequencies in Light group; (**G**–**H**) *Cry1* and *cry2* mRNA, displaying obvious time-phase shift and significantly suppressed oscillation amplitudes in Light group; (**I**) *Rev-erbα* mRNA, showing an obvious time-phase shift in Light group.

In order to elaborate the effect of white LED light on circadian rhythm of clock genes, we examined the mRNA expression profile of clock genes every 6 h for 48 h. In general, LED light exposure disrupted circadian rhythm of clock genes, in a gene-specific manner (Figure 4). Among 9 genes detected, clock (Figure 4A), bmal1 (Figure 4B), cry1 (Figure 4G) and cry2 (Figure 4H) genes displayed obvious time-phase shift and significantly suppressed (*p* < 0.01) oscillation amplitudes, while per1 (Figure 3D), per2 (Figure 4E) and per3 (Figure 4F) genes showed a delayed appearance of the 2^nd^ peak, indicating reduced frequency of oscillation. Interestingly, rorα (Figure 4C) completely lost its circadian rhythm and kept increasing (*p* < 0.01) over the period of examination.

### White LED light disrupts the circadian rhythm of autophagy genes in HT-22 cells

**Figure 5:**
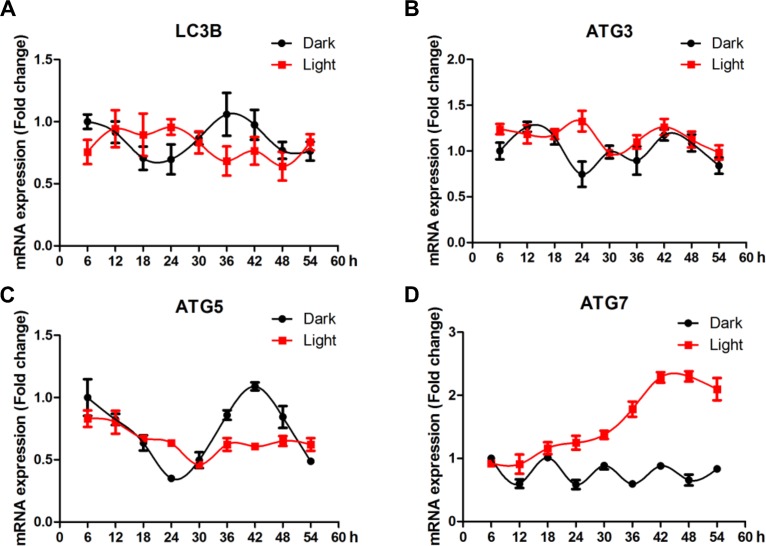
**White LED light influences the circadian rhythm of autophagy-related genes expression in HT-22 cells.** (**A**) *Lc3b* mRNA, with no clear pattern of circadian rhythm and no significant alterations in Light group; (**B**–**C**) *Atg3* and *atg5* mRNA, showing significantly blunted fluctuation in light-exposed cells; (**D**) *Atg7* mRNA, showing a continuous increase in mRNA abundance until a plateau was reached 42 h after light exposure.

Compared to clock genes, autophagy-related genes showed less clear pattern of circadian rhythms (Figure 5). No significant effect of LED light exposure was observed for LC3B (Figure 5A), yet the fluctuation of ATG3 (Figure 5B) and ATG5 (Figure 5C) was significantly blunted (*p* < 0.05) in light-exposed cells. Interestingly, ATG7 (Figure 5D) was significantly up-regulated (*p* < 0.01), reaching a plateau at 42 h after light exposure.

### RORα inhibitor rescues autophagy and normalizes the expression of autophagy-related proteins, but not GR or GSK-3

**Figure 6:**
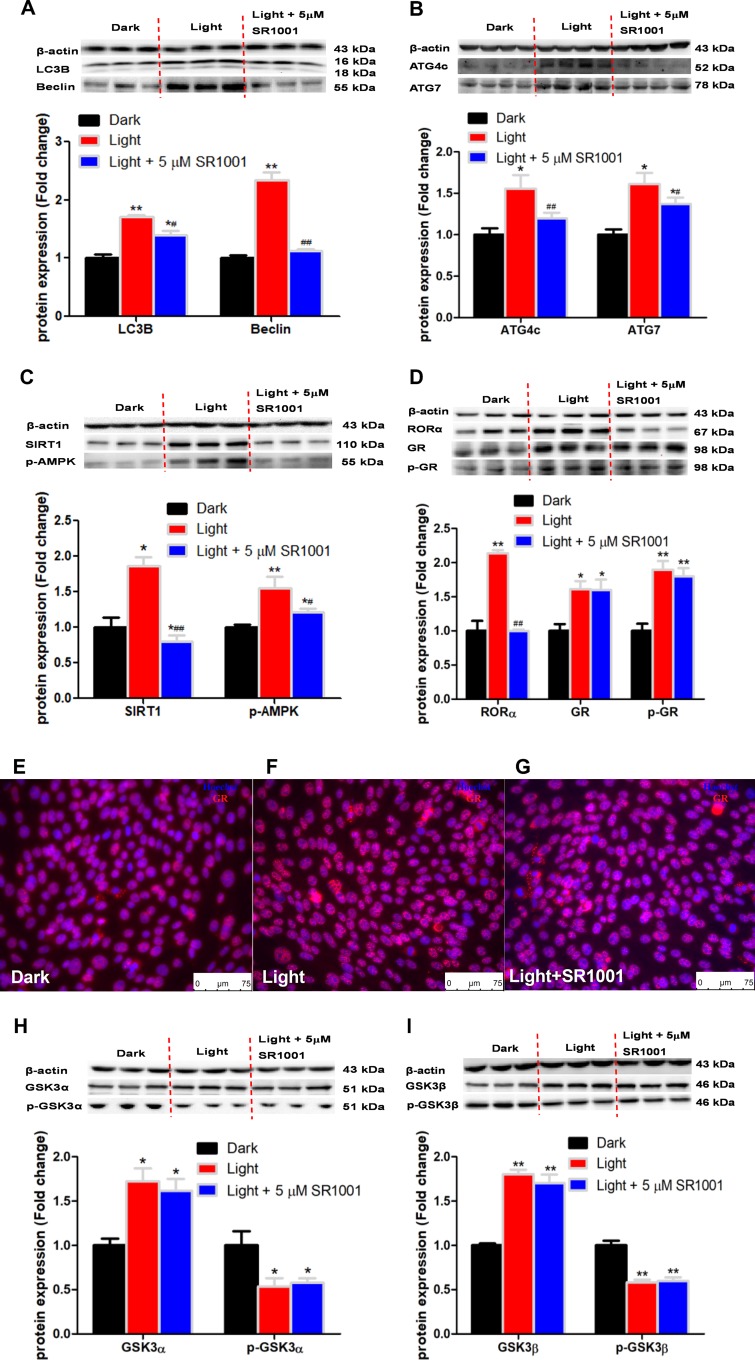
**RORα inhibitor, SR1001, rescues the expression of autophagy-related proteins, yet not GSK3α, GSK-3β and GR in light-exposed HT-22 cells.** (**A**–**D**) Protein content of LC3B, Beclin, ATG4c, ATG7, SIRT1, phospho-AMPK, RORα, GR and phospho-GR. RORα inhibitor SR1001 completely rectified light-induced up-regulation of RORα and autophagy-related proteins, including LC3B, Beclin, ATG4c, ATG7, SIRT1 and phospho-AMPK, in HT-22 cells. However, increases in GR and phospho-GR protein expression were not rectified by SR1001. Values are means ± SEM, *p < 0.05, **p < 0.01, compared with Dark group, ^#^*p* < 0.05, ^##^*p* < 0.01, compared with Light group (n = 3 or 4); (**E**–**G**) Immunofluorescence of GR, showing that SR1001 was not able to restore light-induced GR activation and GR nuclear translocation. The nuclei were stained with Hoechst (blue) and GR was stained with GR antibody (red). Scale bars, 75 μm; (**H**–**I**) Protein content of total and phospho-GSK-3α/β (Ser21/9). Light-induced increase in total GSK-3α/β and decrease in phospho-GSK-3α/β were not rescued by SR1001. Values are means ± SEM, *p < 0.05, **p < 0.01, compared with Dark group (n = 3).

SR1001, a specific RORα inhibitor, significantly rescued the expression of autophagy-related proteins such as LC3B, Beclin (Figure 6A), ATG4c and ATG7 (Figure 6B) in light-exposed HT-22 cells. Accordantly, the light-induced increase in the protein content of SIRT1 and phospho-AMPK was also rescued by SR1001 (Figure 6C). However, SR1001 was not able to restore light-induced GR activation (Figure 6D) and GR nuclear translocation (Figure 6E–Figure 6G). Moreover, SR1001 also failed to rescue the activity of GSK-3α (Figure 6H) and GSK-3β (Figure 6I) in light-exposed cells. These results indicate that RORα plays a pivotal role in white LED light-induced cell autophagy downstream of GR and GSK-3 action.

### GR inhibitor rescues autophagy and the expression of autophagy-related proteins, but not GSK3

**Figure 7:**
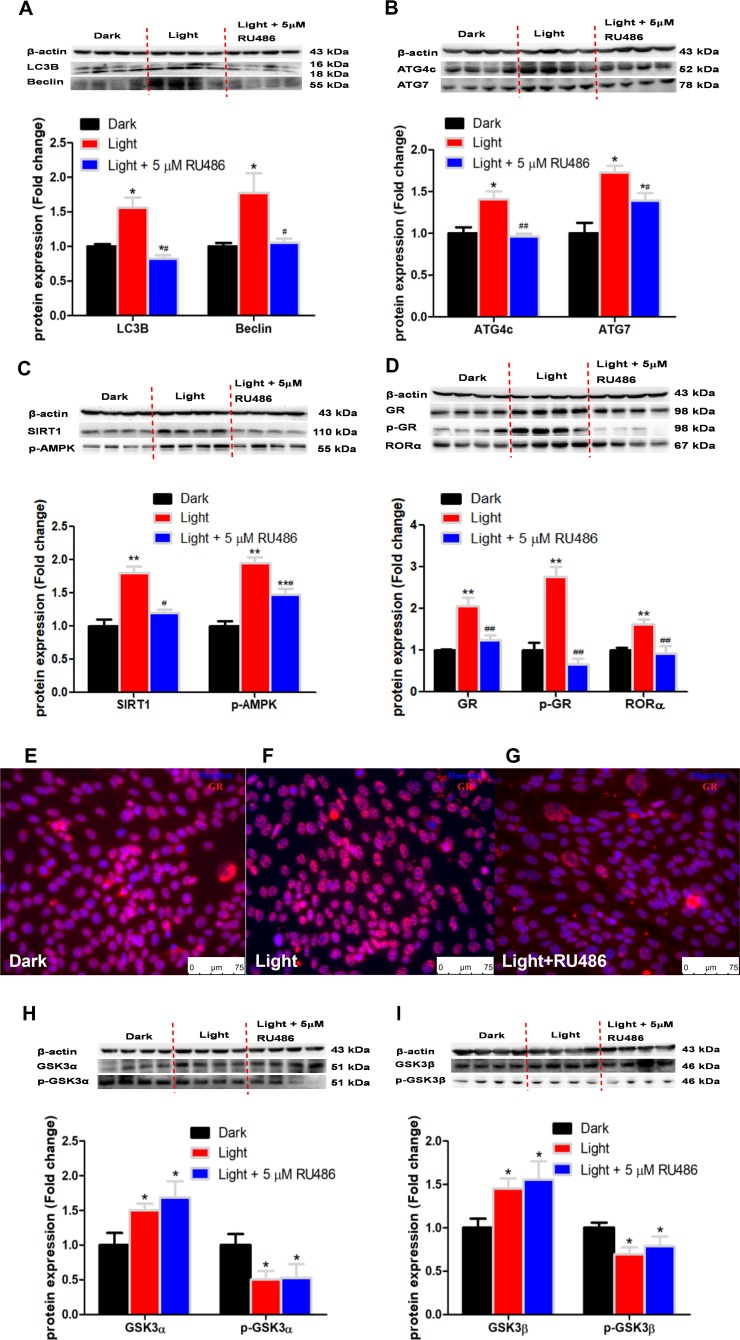
**GR inhibitor, RU486, rescues the expression of autophagy-related proteins and nuclear receptor RORα expression, yet not GSK3α and GSK-3β in light exposure cells.** (**A**–**D**) Protein content of LC3B, Beclin, ATG4c, ATG7, SIRT1, phospho-AMPK, RORα, GR and phospho-GR. GR inhibitor RU486 completely rectified light-induced up-regulation of GR, phospho-GR, RORα and autophagy-related proteins, including LC3B, Beclin, ATG4c, ATG7, SIRT1 and phospho-AMPK, in HT-22 cells. Values are means ± SEM, *p < 0.05, **p < 0.01, compared with Dark group, ^#^*p* < 0.05, ^##^*p* < 0.01, compared with Light group (n = 4); (**E**–**G**) Immunofluorescence of GR, showing that RU486 was able to restore light-induced GR activation and GR nuclear translocation. The nuclei were stained with Hoechst (blue) and GR was stained with GR antibody (red). Scale bars, 75 μm; (**H**–**I**) Protein content of total and phospho-GSK-3α/β (Ser21/9). Light-induced increase in total GSK-3α/β and decrease in phospho-GSK-3α/β were not rescued by RU486. Values are means ± SEM, *p < 0.05, compared with Dark group (n = 4).

GR inhibitor RU486 significantly rescued the expression of autophagy-related proteins such as LC3B, Beclin (Figure 7A), ATG4c and ATG7 (Figure 7B), together with restored protein content of SIRT1 and phospho-AMPK (Figure 7C) in light-exposed HT-22 cells. Moreover, RU486 significantly prevented light-induced up-regulation of RORα protein expression (Figure 7D) and GR activation indicated by GR nuclear translocation (Figure 7E–Figure 7G). However, the increase of GSK-3α and GSK-3β protein expression and the decrease of their phosphorylations induced by LED light exposure were not affected by RU486 (Figure 7H and Figure 7I). These results indicate that GR and RORα play an important role in white LED light-induced autophagy downstream of GSK-3 action.

### GSK-3 inhibitor or knockdown rescues autophagy and activation of autophagy-related proteins as well as GR and RORα expression

**Figure 8:**
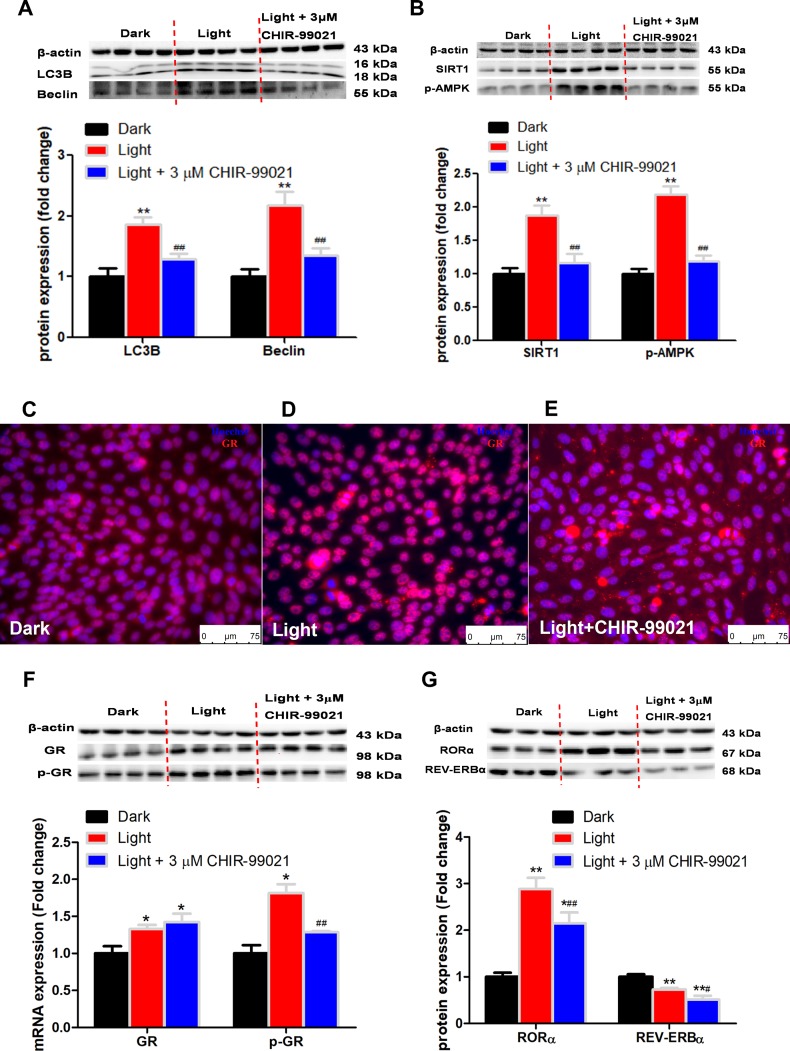
**GSK3 inhibitor, CHIR-99021, rescues the expression of autophagy-related proteins and nuclear receptor GR, RORα expression in light exposure cells.** (**A**–**B**) Protein content of LC3B, Beclin, SIRT1 and phospho-AMPK. GSK3 inhibitor CHIR-99021 completely rectified light-induced up-regulation of autophagy-related proteins, including LC3B, Beclin, SIRT1 and phospho-AMPK, in HT-22 cells. Values are means ± SEM, **p < 0.01, compared with Dark group, ^##^*p* < 0.01, compared with Light group (n = 4); (**C**–**E**) Immunofluorescence of GR, showing that CHIR-99021 was able to restore light-induced GR activation and GR nuclear translocation. The nuclei were stained with Hoechst (blue) and GR was stained with GR antibody (red). Scale bars, 75 μm; (**F**–**G**) Protein content of GR, phospho-GR, RORα and REV-ERBα. CHIR-99021 significantly alleviated light-induced increase of phospho-GR and RORα protein expression, yet the increase of total GR and the decrease of REV-ERBα in Light group were not restored. Values are means ± SEM, *p < 0.05, **p < 0.01, compared with Dark group, ^#^*p* < 0.05, ^##^*p* < 0.01, compared with Light group (n = 3 or 4).

**Figure 9:**
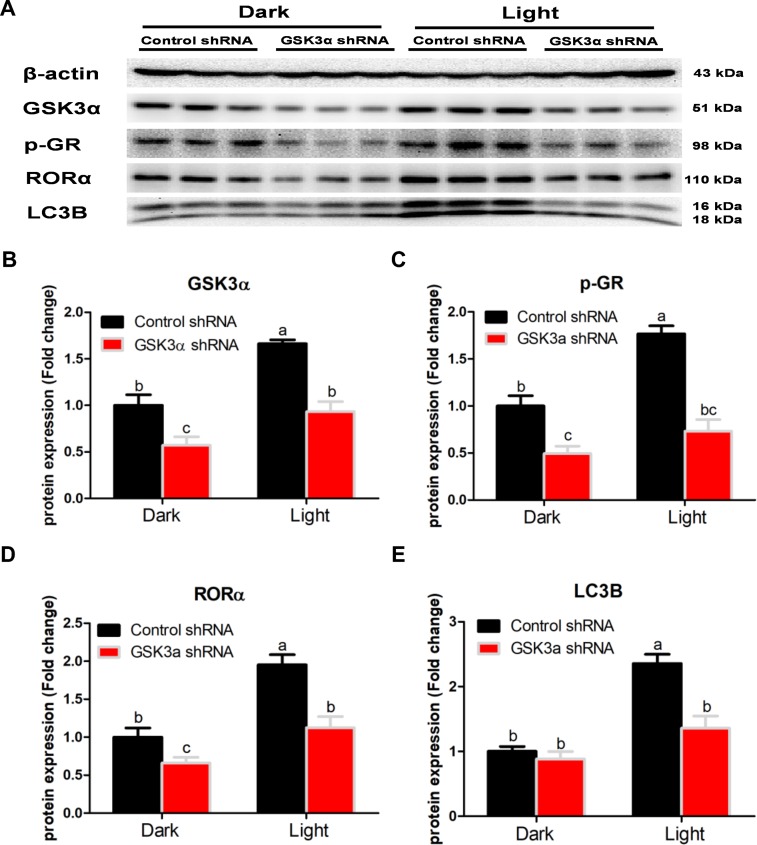
**Knockdown of GSK3α significantly suppresses protein content of GSK3α, p-GR, RORα and LC3B in HT-22 cells.** (**A**) Images of bands detected in Western blot analyses; (**B**) Protein content of GSK3α; (**C**) Protein content of phospho-GR; (**D**) Protein content of RORα; (**E**) Protein content of LC3B. Values are means ± SEM. Bars with different superscripts are significantly different from each other (*p* < 0.05, n = 3).

CHIR-99021, a specific GSK-3 inhibitor, significantly rescued the expression of autophagy-related proteins such as LC3B, Beclin (Figure 8A), together with decreased SIRT1 and phospho-AMPK (Figure 8B) protein expression by CHIR-99021. Accordingly, GSK-3 inhibitor significantly suppressed nuclear translocation of GR in light-exposed cells (Figure 8C–Figure 8E), which was associated with normalized phospho-GR but not total GR protein content (Figure 7F). Moreover, GSK-3 inhibitor partially yet significantly alleviated light-induced increase of RORα protein expression, yet REV-ERBα protein content was further decreased (Figure 8G). These results indicate that GSK-3 serves as an upstream regulator of GR and RORα signaling in autophagy induced by white LED light exposure. This was further supported by shRNA-mediated GSK-3α knockdown (Figure 9A and 9B), which led to significantly blunted or diminished (*p* < 0.01) responses of phospho-GR (Figure 9A and 9C), RORα (Figure 9A and 9D) and LC3B (Figure 9A and 9E) protein expression in light-exposed cells.

## DISCUSSION

A number of studies indicate that constant light exposure significantly suppresses the circadian rhythm of clock-related genes [31, 32], which is associated with impaired hippocampal neurogenesis and cognitive performance [33, 34] in mice. In this study, we found that white LED light exposure significantly disrupted the rhythmic expression of clock-related genes. Interestingly, different genes responded to light exposure differently. Genes such as *clock*, *bmal1*, *cry1* and *cry2*, displayed time-phase shift and suppressed oscillation amplitudes, while others (*per1*, *per2* and *per3*) demonstrated reduced frequency of oscillation. These results agree with previous reports that UVB exposure significantly altered the circadian rhythm of *clock* gene in human keratinocytes [35] and blue light irradiation disrupted the circadian rhythm of *per1*, *per2*, *per3, cry1* and *cry2* genes in Puffer Fish-derived Fugu eye cells [36]. In this study, we observed a striking light response in *rorα* gene that completely lost its circadian rhythm and kept increasing over the period of examination. Similar result was reported that prenatal exposure to continuous light significantly increased *rorα* mRNA expression in the adult rat offspring [37]. Concurrently, the autophagy-related gene *atg7* showed a similar pattern, which is significantly up-regulated reaching a plateau 42 h after light exposure. These findings implicate a possible role of RORα in light-induced autophagy in HT-22 cells.

RORα has been reported to play an important role in the regulation of circadian rhythm [38]. In this study, we found that white LED light exposure significantly up-regulated RORα protein expression in HT-22 cells in association with activation of SIRT1/AMPK signaling, a classical pathway involved in autophagy [39]. RORα inhibitor SR1001 was able to normalize the light-induced autophagy and the expression of autophagy-related proteins. Our results agree with previous report that RORα deprivation results in autophagy dysfunction by interrupting autophagosome clearance in myocardial ischemia/reperfusion injury mice model [40]. Complex interactions exist between the circadian and glucocorticoid systems [41]. In the present study, white LED light significantly induced GR phosphorylation and nuclear translocation. GR antagonist RU486 significantly suppressed autophagy as well as RORα protein expression. Considering that RORα inhibitor SR1001 was not able to restore light-induced GR activation, we presume that GR is up-stream of RORα in the regulation of light-induced autophagy in HT-22 cells. Nevertheless, an in-depth molecular mechanism of GR-mediated RORα activation awaits further investigation. Given that GR activation in response to light exposure in HT-22 cells is ligand-independent, we speculate that there may be a kinase involved in the regulation of GR activation.

GSK-3 is a multifunctional kinase with a critical role in the regulation of autophagy. Previous studies reported that inhibition of GSK-3 activates autophagy in human pancreatic cancer cells [27], breast tumor cell line MCF7 [28], and prostate cancer cells [29]. Contradictory findings are also reported that inhibition of GSK-3β suppresses autophagic cell death in adult hippocampal neural stem cells [26]. The kinase activity of GSK-3 is inhibited through phosphorylation of a serine residue located at the N-terminus of the proteins, S21 and S9 of GSK-3α and GSK-3β, respectively [30]. In the present study, the protein expression of total GSK-3α and GSK-3β was significantly increased, while that of phosphor-GSK-3α (Ser^9^) and phosphor-GSK-3β (Ser^9^) was decreased in response to white LED light exposure, indicating enhanced GSK-3 activity. Inhibition of GSK-3 with CHIR-99021 or shRNA was able to rescue the LED light-induced cell autophagy associated with normalized protein expression of LC3B and Beclin. Interestingly, CHIR-99021 and GSK-3α shRNA also restored the protein expression of phospho-GR and RORα, while light-induced GSK activation was not normalized by RORα or GR inhibition. These results imply that GSK is up-stream of GR and RORα activation induced by white LED light exposure. Our findings are consistent with a previous study that GSK-3 phosphorylates GR on Ser404 and promotes GR activity [42].

In conclusion, the present study provides the first evidence that 24 h exposure under the white LED light induces cell autophagy with disrupted circadian rhythm of clock-related and autophagy-related genes in HT-22 cells. A signaling cascade of GSK-3-mediated GR and RORα pathways is delineated underlying such effects. Our findings indicate that the effects of light exposure in our daily life or in therapy can be detrimental. Caution should be taken to avoid light pollution that has significant impacts on hippocampal neuron survival and related health consequences.

## MATERIALS AND METHODS

### Cell culture and LED exposure

Mouse hippocampal neuron cells (HT-22) purchased from Shanghai HuiYing Biological Technology Co. Ltd. (Shanghai, China) were cultured in Dulbecco’s Modified Eagle’s Medium (DMEM) (Lot AAH204831, SH30243.01, Hyclone) containing 10 % (v/v) FBS at 37°C under 5% CO_2_. Cells were divided into Dark and Light groups and cultured in constant dark or light (2500 lux white LED) condition for 24 h. The white LED light was purchased from Wangdeng Technology Co., LTD (ShenZhen, China). The CCT of the light source was 6500 K (Light spectrums in Supplementary Figure 1) and the surface power density was 15 mW/cm^2^. After cultured under respective condition for 24 h, cells were harvested and the culture media were collected.

### Serum shock procedures

Approximately 5 × 10^5^ cells/well were seeded in 6-well plates and cultured in DMEM containing 10 % (v/v) FBS at 37°C under 5% CO_2_ for 24 h before the medium was replaced with serum-rich medium (DMEM, supplemented with 50% horse serum, Hyclone) for serum shock. Two hours after the serum shock (time = 0), the medium was replaced with serum-free DMEM and the cells were divided into Dark and Light groups. Cells were collected every 6 h to 54 h after light treatment. At the indicated times, the cells were washed twice with ice-cold PBS, frozen on a layer of liquid nitrogen, and kept at -80°C until the extraction of whole cell RNA.

### Total RNA isolation and real-time PCR

Total RNA was extracted from the HT-22 cells using 700 μL TRIzol reagents (Cat#3101-100, Invitrogen, USA). Two micrograms of total RNA were treated with RNase-Free DNase and reverse-transcribed to cDNA using the HiScript^®^ Q RT SuperMix (Vazyme, China). Four microliters of diluted cDNA (1:25, vol/vol) were used for real-time PCR performed in Mx3000P (Stratagene, USA). Peptidylprolyl isomerase A (PPIA) was used as an internal control to normalize the technical variations. Data were analyzed using the method of 2^–ΔΔCT^ and presented relative to the Dark group. Nucleotide sequences of specific primers are shown in Table 1.

**Table 1:** Nucleotide sequences of specific primers.

**Target genes**	**Primer sequences (5’ to 3’)**		**GenBank accession**
lc3b	F: TAACCAAGCCTTCTTCCTCC	R: GCTGTCCCGAATGTCTCC	NM_026160
atg3	F: AACAAGAACATACGACCTG	R: GCTCATCATAGCCAAAC	NM_001356366
atg4c	F: CTGGCGATTGGTATGGA	R: CACGGAGTCAGTCTGCTTA	NM_175029
atg5	F: CAAGGATGCGGTTGAGGC	R: TGAGTTTCCGGTTGATGG	NM_053069
atg7	F: CCTCGCTGGGACTTGTG	R: TGAATCCTTCTCGCTCGT	NM_001253717
beclin	F: GGCCAATAAGATGGGTCTGA	R: GCTGCACACAGTCCAGAAAA	NM_001359820
clock	F: TCACTCAGGACAGACAGAT	R: TGGCGAAGGTAGGATAGG	NM_007715
bmal1	F: GCTGGACGAAGACAATGA	R: AAGTTCCTGTGGTAGATACG	NM_007489
cry1	F: ATCTTGATGCCAATCTACGA	R: AGTGATGTTCCATTCCTTGA	NM_007771
cry2	F: TGTCCCTTCCTGTGTGGAAGA	R: GCTCCCAGCTTGGCTTGA	NM_009963
per1	F: ACCTCTGGCTGTTCCTAC	R: CCTCTGCTTGTCATCATCA	NM_011065
per2	F: ATGCTCGCCATCCACAAGA	R: GCGGAATCGAATGGGAGAAT	NM_011066
per3	F: CTCTGGCTTCTGAACATACT	R: TCATACTGCGAGGCTCTT	NM_001289878
nr3c1	F: AGCAGTGGAAGGACAGCAC	R: GTAGGGGTAAGCTGTGGCAG	NM_008173
rorα	F: ATGCACCTGACCGAAGACGAA	R: AGCTTTTCCGTATGTCGTCCAC	NM_013646
rev-erbα	F: GAAGTGTCTCTCCGTTGGCA	R: CTGCTCAGTTGGTTGTTGGC	NM_007489
gsk3α	F: GCTTCTCCCCTCACCACTTC	R: AGGGTAGCAGTTGTGGCATC	NM_001031667
gsk3β	F: GAGCAGGACATTTCACCCCA	R: AAGAGTGCAGGTGTGTCTCG	NM_001347232
ppia	F: GCAAGACCAGCAAGAAGA	R: CAGTGAGAGCAGAGATTACA	NM_008907

### Total protein extraction and Western blotting

Whole cell lysates were extracted and the protein concentration was determined using Pierce BCA Protein Assay kit (Rockford, IL, USA) according to the product instruction. Whole cell proteins (30 µg) were loaded on a 10% SDS-PAGE gel for electrophoresis. Western blot analysis for LC3B (AP0762, Bioworld, USA, diluted 1:500), Beclin (AP0769, Bioworld, USA, diluted 1:500), ATG4c (5262, Cell Signaling Technology, USA, diluted 1:1000), ATG7 (2631, Cell Signaling Technology, USA, diluted 1:1000), GSK-3α (9338, Cell Signaling Technology, USA, diluted 1:1000), phospho-GSK-3α (9316, Cell Signaling Technology, USA, diluted 1:1000), GSK-3β (9315, Cell Signaling Technology, USA, diluted 1:500), phospho-GSK-3β (9336, Cell Signaling Technology, USA, diluted 1:500), GR (ab2768, Abcam, USA, diluted 1:500), phospho-GR (4161, Cell Signaling Technology, USA, diluted 1:1000), RORα (ab60134, Abcam, USA, diluted 1:500), REV-ERBα (ab174309, Abcam, USA, diluted 1:500), SIRT1 (ab12193, Abcam, USA, diluted 1:1000), AMPK (BS1009, Bioworld Technology USA, diluted 1:1000), phospho-AMPK (2531, Cell Signaling Technology, USA, diluted 1:1000), SQSTM1/p62 (bs-2951R, Bioss, USA, diluted 1:1000), was carried out according to the recommended protocols provided by the manufacturers, and β-actin (AP0060, Bioworld, USA, diluted 1:10,000) was used as loading control. Images were captured by VersaDoc 4000MP system (Bio-Rad, USA) and the band density was analyzed with Quantity One software (Bio-Rad, USA).

### Measurement of autophagosome formation

HT-22 cells were seeded on coverslips in 12-well plates, and grown to 70% to 80% confluency before a Lipofectamine 2000-mediated transfection with the green fluorescent protein (GFP)-tagged microtubule-associated protein 1 light chain 3 beta (MAP1LC3B) expression plasmid (GFP-MAP1LC3B), which was a gift from Prof. Honglin Liu (Nanjing Agricultural University). Cells were cultured for 48 h before exposed to light treatments, and the cytoplasmic GFP-MAP1LC3B puncta were then observed under a laser-scanning confocal microscope (Carl Zeiss, Zeiss LSM 710 META). Experiments were performed in triplicate, and the punctate GFP-MAP1LC3B was counted in 3 randomly selected fields in each coverslip.

### Determination of autophagy flux

To detected the effect of white LED light on autophagy flux, HT-22 cells were treated with 50 μM chloroquine (CQ, C6628, sigma, USA), a specific protease inhibitor, in the last 2 h of white LED light exposure. Then the levels of autophagy related protein LC3B and p62, as well as both the net and relative autophagy flux of LC3B and p62 were detected.

### Transmission electron microscopy

HT-22 cells were treated with typsin for 5 min at 37 °C and centrifuged at 200 ×g for 5 min. After the supernatant was removed, the cell pellets were fixed in 2% glutaraldehyde in 0.1 M sodium cacodylate (NaCac) buffer, pH 7.4, postfixed in 2% osmium tetroxide in NaCac, stained en bloc with 2% uranyl acetate, dehydrated in a graded ethanol series, and embedded in Epon-Araldite resin. Thin sections were made using a diamond knife on a Leica EM UC6 ultramicrotome (Leica Microsystems, Wetzlar, Germany), collected on copper grids, and stained with uranyl acetate and lead citrate. Cells were observed under a JEM 1230 transmission electron microscope (JEOL USA, Peabody, MA) at 110 kV and imaged with an UltraScan 4000 CCD camera and First Light Digital Camera Controller (Gatan, Pleasanton, CA). Autophagic vacuoles were counted in individual cells from multiple fields and nonserial sections.

### Determination of GR nuclear translocation

Sub-cellular localization of GR protein in HT-22 cells was determined through immunofluorescence staining analysis. After 24 h culture, HT-22 cells were fixed with 4% paraformaldehyde for 10 min and treated with 0.3% TritonX-100 for 30 min on coverslips. After washing with PBS for three times and blocking with 10% Fetal Bovine Serum for 10 min, HT-22 cells were incubated with GR antibodies (ab2768, Abcam, USA, diluted 1:500) at 4°C overnight. Signal of primary antibody was amplified by TRITC-labelled goat anti-rabbit IgG (ab97200, Abcam, USA, diluted 1:1000) and were visualized and imaged with a fluorescence microscope.

### Knockdown of GSK-3α by shRNA transfection

To analyze the function of GSK-3α, HT-22 cells were transfected with a shRNA plasmid constructed to specifically knockdown GSK-3α (pPLK/GFP+Puro-Gsk3a shRNA-1). HT-22 cell line was maintained at 37°C in a humidified incubator containing 5% CO_2_. Cells were grown in medium supplemented with 10% FBS in 6-well plates 24 h prior to transfection and the medium was replaced by fresh DMEM immediately before transfection. The transfection was conducted following the protocols of Lipofectamine 2000 Transfection Reagent (11668-019, Invitrogen, US) and Opti-MEM (31985, Gibco, USA) provided by the manufacturers.

### Statistical analysis

All data are presented as means ± SEM and the differences between groups were analyzed using independent-samples T-Test with SPSS 20.0 for Windows. The differences were considered statistically significant when *P* < 0.05.

## Supplementary Material

Supplementary Figures
