# Supplementary material for: White light emitting diode induces autophagy in hippocampal neuron cells through GSK-3-mediated GR and RORα pathways
Source: Aging (Albany NY). 2019 Mar 28;11(6):1832–49. doi: 10.18632/aging.101878 (PMC6461168; doi:10.18632/aging.101878)
Supplement: Supplementary Figures [file aging-11-101878-s001.pdf]

SUPPLEMENTARY MATERIALS

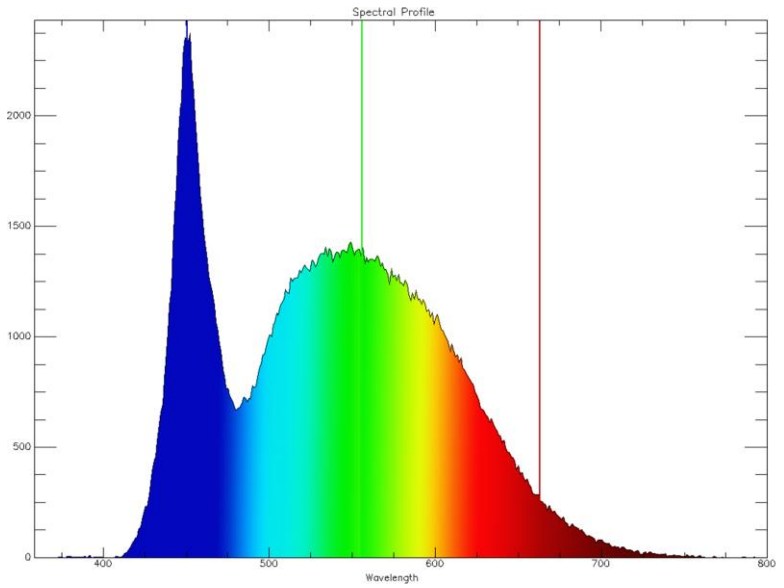

Supplementary Figure 1. Light spectrum of multichromatic white LED light.

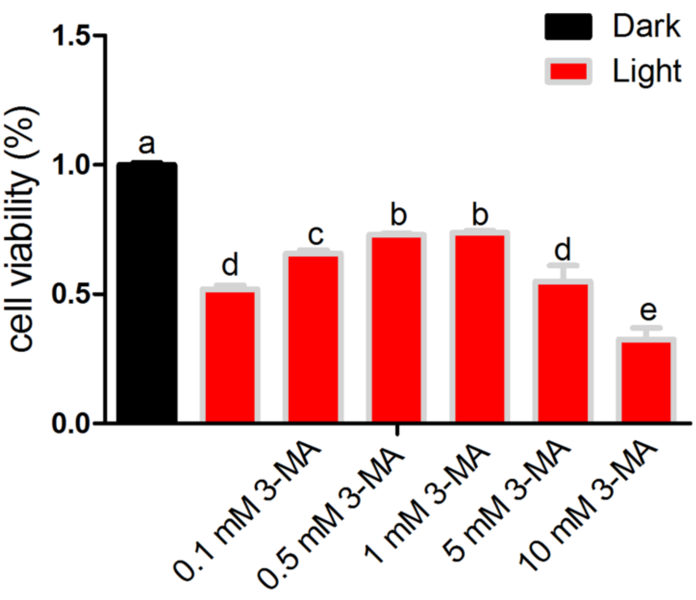

Supplementary Figure 2. Cell viability in LED light exposed HT-22 cells treated with different concentrations of autophagy inhibitor, 3-MA. At low concentrations up to 1 mM, 3-MA was able to partially rescue the light-induced decrease in cell viability, yet at high concentrations, 5 mM and 10 mM, 3-MA failed to rescue or even further reduced the cell viability. Values are means ± SEM. Bars with different superscripts are significantly different from each other ($p < 0.05$, n = 10).

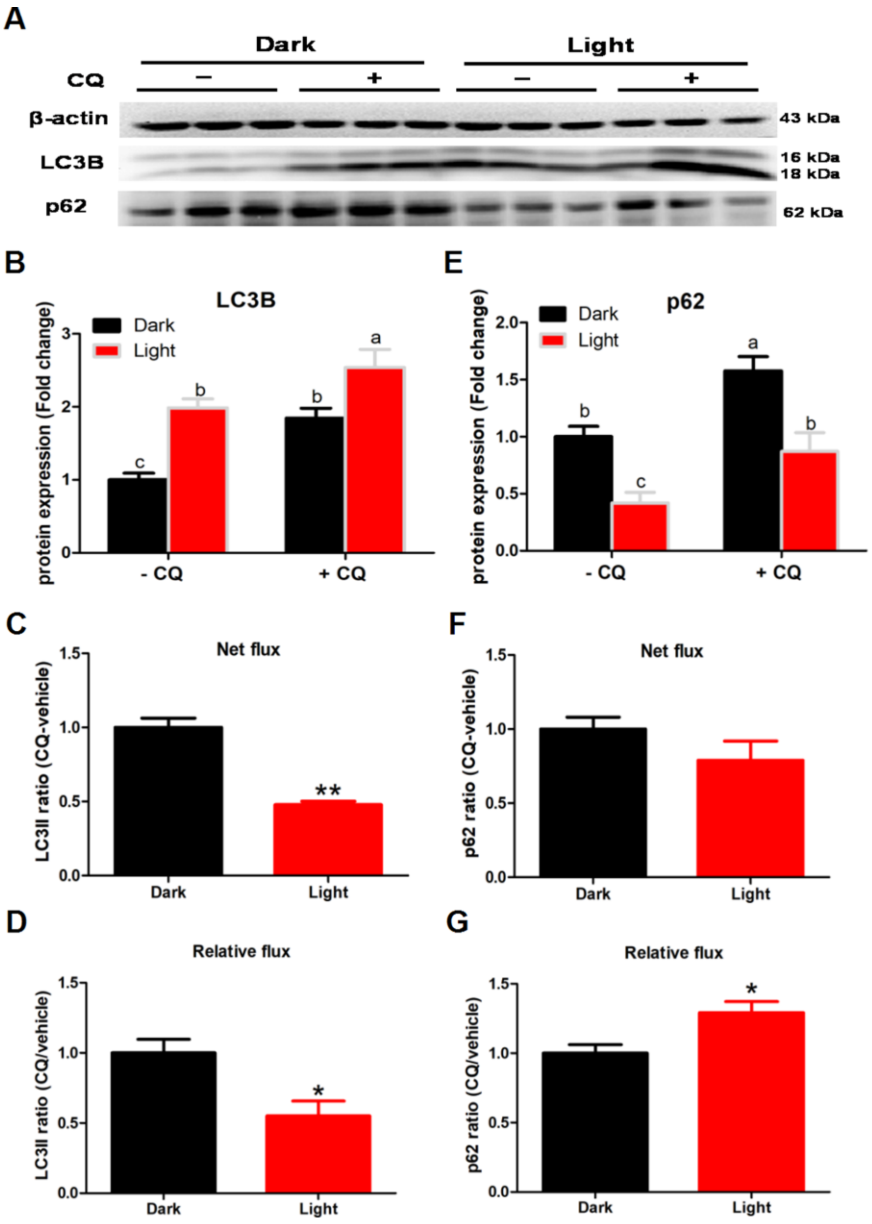

Supplementary Figure 3. White LED light exposure influence autophagy flux in hippocampal neuron cells. To detected the effect of white LED light on autophagy flux, we added 50 μM CQ with 2h after white LED light exposure. (**A**) Images of bands detected in Western blot analyses; (**B**) LC3B protein levels in Dark and Light group with or without CQ. Values are means ± SEM. Bars with different superscripts are significantly different from each other ($p < 0.05$, n = 3); (**C**) Net flux of LC3II protein. Values are means ± SEM, ** $p < 0.01$ compared with Dark group, n = 3; (**D**) Relative flux of LC3II protein. Values are means ± SEM, * $p < 0.05$ compared with Dark group, n = 3; (**E**) p62 protein levels in Dark and Light group with or without CQ. Values are means ± SEM. Bars with different superscripts are significantly different from each other ($p < 0.05$, n = 3); (**F**) Net flux of p62 protein. Values are means ± SEM, n = 3; (**G**) Relative flux of p62 protein. Values are means ± SEM, * $p < 0.05$ compared with Dark group, n = 3.
